# Expanded access with intravenous hydroxypropyl-β-cyclodextrin to treat children and young adults with Niemann-Pick disease type C1: a case report analysis

**DOI:** 10.1186/s13023-019-1207-1

**Published:** 2019-10-21

**Authors:** Caroline Hastings, Camilo Vieira, Benny Liu, Cyrus Bascon, Claire Gao, Raymond Y. Wang, Alicia Casey, Sharon Hrynkow

**Affiliations:** 1 Department of Pediatric Hematology Oncology, UCSF Benioff Children’s Hospital Oakland, 747 52nd Street, Oakland, CA 94609-1809 USA; 2 Department of Pediatrics, University of California San Francisco, San Francisco, CA USA; 3 Universidade Federal da Bahia, Clínica Citta, Ed. Mundo Plaza, Av. Tancredo Neves, 620, Sala 1905, Camino dos Árvares, Salvador, Brazil; 4 GI & Liver Clinics, Highland Hospital, Alameda Health System, Highland Hospital, Oakland, CA USA; 5 Division of Gastroenterology & Hepatology, Highland Hospital, Alameda Health Systems, Highland Care Pavilion 5th floor, 1411 East 31st Street, Oakland, CA 94602 USA; 6 UCSF Benioff Children’s Hospital Oakland, Oakland, CA USA; 7 Present Address: Neuroscience Graduate Program, Brown University, 185 Meeting Street, Box GL-N, Providence, RI 02912 USA; 8 Division of Metabolic Disorders, Children’s Hospital of Orange County, CHOC Children’s Specialists, 1201 W. La Veta Ave, Orange, CA 92868 USA; 9 Department of Pediatrics, University of California, Irvine School of Medicine, Irvine, CA 92868 USA; 10 Boston Children’s Hospital, 300 Longwood Avenue, Boston, MA 02115 USA; 11 CTD Holdings, Inc., P.O. Box 1180, Alachua, FL 32616 USA

**Keywords:** Niemann-pick disease type C, Hydroxypropyl-beta-cyclodextrin, Intravenous administration, Investigational new drug, hepatomegaly, splenomegaly, lung disease

## Abstract

**Background:**

Niemann-Pick Disease Type C (NPC) is an inherited, often fatal neurovisceral lysosomal storage disease characterized by cholesterol accumulation in every cell with few known treatments. Defects in cholesterol transport cause sequestration of unesterified cholesterol within the endolysosomal system. The discovery that systemic administration of hydroxypropyl-beta cyclodextrin (HPβPD) to NPC mice could release trapped cholesterol from lysosomes, normalize cholesterol levels in the liver, and prolong life, led to expanded access use in NPC patients. HPβCD has been administered to NPC patients with approved INDs globally since 2009.

**Results:**

Here we present safety, tolerability and efficacy data from 12 patients treated intravenously (IV) for over 7 years with HPβCD in the US and Brazil. Some patients subsequently received intrathecal (IT) treatment with HPβCD following on average 13 months of IV HPβCD. Several patients transitioned to an alternate HPβCD. Moderately affected NPC patients treated with HPβCD showed slowing of disease progression. Severely affected patients demonstrated periods of stability but eventually showed progression of disease. Neurologic and neurocognitive benefits were seen in most patients with IV alone, independent of the addition of IT administration. Physicians and caregivers reported improvements in quality of life for the patients on IV therapy. There were no safety issues, and the drug was well tolerated and easy to administer.

**Conclusions:**

These expanded access data support the safety and potential benefit of systemic IV administration of HPβCD and provide a platform for two clinical trials to study the effect of intravenous administration of HPβCD in NPC patients.

## Introduction

Niemann-Pick disease, Type C (NPC) is a pan-ethnic, often fatal, autosomal recessive lysosomal storage disease characterized clinically by peripheral organ dysfunction, psychiatric manifestations, and neurodegeneration [1, 2]. Reported incidence is approximately 1 in 90,000-120,000 [3–6]. Loss of function mutations in the *NPC1* or *NPC2* genes cause a cholesterol transport defect which results in the accumulation of lipids (cholesterol, sphingomyelin, sphingosine and glycosphingolipids) within the endosome. Toxic accumulation of cholesterol and these other lipids are responsible for the clinical features of the disease [1, 7–13]. The symptoms associated with NPC vary with the age of onset. These include visceral manifestations (organomegaly, liver and lung dysfunction), a movement disorder (cerebellar ataxia), problems with fine motor manipulation (dystonia, dysmetria), and speech and swallowing (dysarthria and dysphagia). Impaired vertical supranuclear saccades, often leading to complete supranuclear gaze palsy (VSGP), is also a key clinical feature and one of the earliest and most frequently noted signs by patient families and physicians. Impairment of vertical saccades occurs early in the disease and development and slowing of horizontal saccades correlate with disease progression and possibly severity and may be an important risk indicator [14–20]. Seizures as well as cataplexy are frequent in patients with progressive neurologic decline [21–23]. Progressive developmental delay and altered memory and cognition are present in many patients. A range of behavioral and/or psychiatric disorders including psychosis, bipolar disease, and attention deficit may be apparent as initial or later manifestations of NPC [1, 5, 14, 24–26].

Diagnosis of NPC is often delayed due to the highly heterogeneous clinical phenotypes, likely due to genotypic polymorphism [24, 25, 27], coupled with lack of awareness of the disease amongst clinicians [19–21]. NPC may present at any age, from the perinatal period up to even the sixth or seventh decade of life [1, 16, 28–31]. Most patients diagnosed with the condition die between 10 and 25 years of age [1, 5, 24].

### Natural history of systemic manifestations of NPC

NPC is classified as a neurovisceral disorder and the neurologic, psychiatric or visceral signs may arise independently of one another and follow a different course of progression [1]. Patients presenting in infancy to early childhood typically have isolated hepatosplenomegaly, which may stay isolated for many years. Older children and adolescents frequently will have splenomegaly either as an isolated early finding on physical exam or the inaugural sign of the disease [1]. Splenomegaly may resolve in patients when neurological symptoms develop or later, but typically 80–90% of patients will have evidence of organomegaly [3, 16].

The severity of neurocognitive involvement often defines the projected outcome, but is typically preceded by signs or symptoms of systemic disease. In a recent epidemiologic study of 53 patients with NPC in the United Kingdom, 17 (32%) had a systemic presentation [24]. Systemic manifestations include neonatal cholestatic jaundice, sometimes progressing to fulminant liver failure, transient jaundice in the newborn period, isolated hepatomegaly or splenomegaly or both, and/or evidence of hypersplenism (decreased blood counts such as thrombocytopenia, leukopenia, or anemia related to enlarged spleen size) [1, 14, 24, 32, 33]. NPC should be strongly suspected in the neonate with cholestasis [34] and is one of several inherited metabolic disorders to be a recognized cause of fatal acute liver failure in the newborn or young child [35]. Though the majority of infants will experience early resolution of jaundice, the organomegaly often remains for a variable period of time and precedes onset of neurologic manifestations. Children with fulminant and rapidly progressive liver disease (approximately 10% of those with cholestasis) typically die within the first 6 months of life and some have undergone liver transplantation [24]. Our report includes a young patient with evidence of severe liver disease who responded favorably to IV HPβCD with decreasing liver size and improved synthetic function.

As the defect in the NPC protein affects every cell of the body it can be assumed all organs would be affected, though not all organs show evidence of irreversible damage or overt clinical significance. Lung disease is extremely rare in NPC, though the pathologic features are not well characterized and it is more prevalent in patients with *NPC2* as compared to *NPC1* [36]. Patients with primary lung involvement show evidence of interstitial lung disease with thickened septa, foamy macrophages and infiltrative leukocytes [37]. A restrictive pattern is evident on pulmonary function testing. Patients with severe neurologic manifestations, with or without lung disease, can develop recurrent aspiration pneumonias, resultant hypoxia and chronic lung disease [38].

Current treatment options for patients with NPC are limited to supportive care measures and the use of miglustat (N-butyl-deoxynojirimycin, Zavesca®), the only disease specific agent approved for the treatment of neurological manifestations of NPC by the European Medicines Agency in 2009. It is not currently FDA approved for use in the United States for this indication [3, 39–46]. Observations from cohort studies and NPC registries suggest miglustat has an impact on stabilization of neurologic symptoms, an effect that appears most pronounced in the juvenile and adult onset groups [20, 39, 41, 42, 44, 47]. Miglustat appears to have no impact on organomegaly or systemic manifestations of disease [20]. The international disease registry for Niemann Pick type C provides the largest data base to date for assessment of safety and response to intervention, with 69% of patients demonstrating improvement/stability utilizing composite disability scores in patients who received continuous miglustat for an average period of 2 years [39]. Assessment of retrospective data collected in registries is subject to variable collection practices, and the low numbers of untreated patients limits conclusions of the effect of miglustat versus standard of care on outcomes. Current licensed options for disease modification are inadequate to address the heterogeneity of the disease including the disease effect on the brain and peripheral organs.

One emerging therapeutic that may target the systemic and neurologic features of NPC is hydroxypropyl-β-cyclodextrin (HPβCD). HPβCD is a cyclic oligosaccharide consisting of seven glucopyranose units, with a hydrophilic exterior and a hydrophobic interior, thus enhancing the solubility of poorly water-soluble compounds (such as cholesterol) via formation of compound-cyclodextrin complexes [48]. As such, HPβCD has been utilized as an excipient facilitating transport of molecules across membranes [49]. Early studies in NPC mice testing allopregnanolone complexed with HPβCD demonstrated prolongation of lifespan [50–52]. The first studies involving NPC mice confirming HPβCD alone delivered systemically was responsible for the beneficial effects on cholesterol metabolism, decreasing total body cholesterol burden, and ameliorating neurological symptoms, opened new therapeutic avenues for NPC patients [53–57]. The first clinical protocol (2009–2010 Hastings/Hempel protocol and FDA reports available online) [58] was developed based on the initial discovery of the positive effects of HPBCD on the NPC mouse model by Dr. Benny Liu at the University of Texas Southwestern, in the lab of Dr. John Dietschy [53]. Prolonged lifespan and clinical benefit were confirmed in subsequent studies following the administration of HPβCD to mouse and feline models of NPC disease [59–61].

We report here a series of 12 case histories of individuals with NPC who have either received intravenous (IV) HPβCD only or IV followed by concurrent IV and intrathecal (IT) HPβCD (herein referred to as sequential or SEQ). The investigators utilized the original Hastings/Hempel protocol for treatment and monitoring guidelines. Some investigators chose to modify the protocol with respect to dosage and/or intervals, as detailed in this report. Two formulations of HPBCD were used, Trappsol® Cyclo™ and Kleptose®. Nine of the patients received Trappsol® Cyclo™ beginning in 2009, four exclusively, and five received this formulation initially followed by a change to Kleptose® HPβCD. For patients receiving both formulations, the vast majority of their experience with HPβCD was with Trappsol® Cyclo™. Three patients received Kleptose® exclusively. The earliest use of Kleptose® was in 2013, when a compounded formulation became available. The FDA allowed interchange of these two products for patients receiving compassionate use HPβCD for NPC and they are therefore referred to only as HPβCD in this publication.

## Methods

The patient families and principal investigators utilizing the Hastings/Hempel expanded access protocol (or adaptation thereof) for IV HPβCD were contacted by the authors (SH and CH) to participate in a data collection protocol. A data acquisition protocol was developed by the lead author (CH) and investigators sought approval from local ethics or Institutional Review Boards. Case Report Forms (CRFs) were developed to capture patient characteristics including manifestations of disease, adverse events (AEs), details of drug administration, laboratory and clinical assessments.

Informed consent was obtained from all of the subjects prior to the initiation of treatment with HPβCD in accordance to the local Institutional Review Boards and principles of ethical research according to the Declaration of Helsinki [62]. Further consent was obtained from the patient families to participate in the data acquisition protocol.

The original Hastings/Hempel protocol was developed in 2008 and granted Investigational New Drug (IND) approval [Hastings C. Compassionate Use of Hydroxy-propyl-ß-cyclodextrin in Identical Twins Suffering from Niemann Pick Type C; IND 104,114 and IND 104,116; submitted to FDA December 1, 2008]. This protocol was utilized internationally by 2009 and included assessments for safety with laboratory and clinical assessments. The first U.S. patients (SEQ 1,2) treated on this protocol initiated intravenous dosing at 80 mg/kg/hour for 4 days as a continuous infusion over 4 days, then transitioned to biweekly dosing over 8 h, reaching a maximum of 2800 mg/kg/dose. Pharmacokinetic data obtained at a dose of 2500 mg/kg IV weekly (data not shown) were submitted to the FDA for review and serum concentrations approximated effective tissue concentration levels (target between 1 and 3 mM), the concentration at which prior mouse studies HPβCD was shown to act as a cholesterol shuttle, transporting cholesterol between membranes. [34, 54, 58, 63] High cellular concentrations (10–100 mM) were avoided, in which HPβCD is known to serve as a cholesterol sink and can extract cholesterol from cellular membranes resulting in cell membrane damage and toxicity [64]. Further, animal studies confirmed a dose limited effect [65, 66]. These PK studies provided the earliest data in determining dosing and intervals for the shared protocol. Intrathecal dosing began 18 months later in these patients, in 2010, at an initial dose of 175 mg every 2 weeks. The dose was increased to 350 mg after a 3-month treatment and safety assessment period. SEQ patients 3,4,5,6 and 9 have followed the protocol as of the 2010 dosing protocol. Pharmacokinetic data were obtained to determine CSF drug concentrations to approximate those observed in the in vitro and in vivo mouse studies (data not shown). Other investigators utilizing this protocol initiated dosing at the same levels and some chose to dose escalate (dosing summarized in Table 1).

**Table 1:** Clinical manifestations, HPβCD treatment data and adverse events

Patient identification	Age at start of IV HPβCD treatment	Symptom progression at start of IV treatment	Intravenous treatment Dose/Interval; Length of treatment	Time interval between start of IV and IT HPβCD treatment	Intrathecal treatment Dose/Interval; Length of treatment	Adverse effects, IV HPβCD	Adverse effects, IT HPβCD
SEQ1	5 years	Ataxia, VSGP, loss of language, dysphagia, global developmental delay	80 mg/kg/day to 2800 mg/kg twice weekly; stable at 2500 mg Q2 weeks; 92 months	18 months	175 mg every 2 weeks; advanced to 350 mg every 2 weeks (IO 50 mg substituted); 74 months	None	Seizures, increased frequency 24 h post IT
SEQ2	5 years	Ataxia, VSGP, loss of language, dysphagia, global developmental delay	80 mg/kg/day to 2800 mg/kg twice weekly; stable at 2500 mg Q2 weeks; 92 months	18 months	175 mg Q2weeks; advanced to 350 mg every 2 weeks (IO 50 mg substituted); 74 months	None	Seizures, increased frequency 24 h post IT; Intracranial hemorrhage secondary to Ommaya insertion, removal of Ommaya
SEQ3	15 years	VSGP, progressive cognitive impairment, seizures, fine motor coordination, psychosis, ataxia	1200 mg/kg with increase over 8 months to 2500 mg/kg weekly; 83 months	16 months	175 mg (advanced to max 875 mg), then stabilized at 350 mg Q15 days; IO 100 to 350 mg every 15 days prior to removal at 10 months; 67 months	Port-a-Cath infection (twice), removal after 2nd infection	Meningitis, removal of Ommaya
SEQ4	11 years	VSGP, progressive cognitive impairment, seizures, fine motor coordination, psychosis, ataxia, gelastic cataplexy	1200 mg/kg with increase over 8 months to 2500 mg/kg weekly; 83 months	16 months	IT advanced from 175 to 875 mg Q15 days; now receives IO 100 mg every 15 days; 67 months	Port-a-Cath infection (twice), removal after 2nd infection	None
SEQ5	13 years	Dysarthria, dysphagia, partial complex seizures, worsening ataxia and VSGP, obstructive sleep apnea	500 mg/kg advanced to 2000 mg/kg twice weekly; 72 months	13 months	350 mg Q2 weeks, advanced to 600 mg Q2 weeks, then dropped to 500 mgQ2 weeks; 59 months ^a^LP	None	Nausea, emesis thought secondary to dehydration; Increased frequency seizures for 24 h post IT; Mild high frequency hearing loss at 500–600 mg
SEQ6	10 years	Splenomegaly, mild VSGP; precocious puberty (not related to NPC)	500 mg/kg advanced to 2000 mg/kg twice weekly; 68 months	10 months	350 mg Q2 weeks, advanced to 500 mg Q2 weeks; 59 months	None	Mild high frequency hearing loss at 500 mg
SEQ7	2 years	Progressive neurocognitive decline, VSGP, lung disease, thrombocytopenia, leukopenia	1500 mg/kg weekly to 2000 mg/kg weekly; 58 months	23 months	150 mg every 2 weeks, dose escalation to 750 mg every 2 weeks; 35 months	Pneumonia, viral illnesses	None reported
SEQ8	21 months	Worsened hepatosplenomegaly, severe growth retardation, tracheomalacia/ bronchomalacia (not related to NPC), tracheostomy, ventilator assist	500 mg/kg weekly, escalated by 500 mg/kg monthly to 2000 mg/kg weekly; 30 months	4 months	175 mg every 4 weeks; dose escalated to 400 mg, then decreased to 300 mg every 2 weeks; 26 months	CVC malfunction; Seizures	Increased seizures frequency for 24 h post IT at higher doses (400 mg)
SEQ9	24 years	Progressive neurocognitive decline, memory impairment, falling, gaze palsy, swallowing problems	2500 mg/kg weekly, transitioned to every 2 weeks; 21 months	1 month	350 mg every 2 weeks; 20 months ^a^LP	Nausea	Nausea
IV1	18 years	Spastic quadriplegia, recurrent pneumonia (tracheostomy, ventilator dependent), dysphagia and enterally fed, refractory seizure disorder	500 mg/kg weekly, escalated by 500 mg/kg monthly to 2000 mg/kg weekly; 17 months	N/A	N/A	Port-a-Cath infection; proteinuria, elevated transaminases 5x baseline; fevers, hypertension	N/A
IV2	27 years	Hepatosplenomegaly, mild thrombocytopenia, severe neurocognitive impairment, wheelchair dependent, VSGP, nasogastric tube fed, severe dysmetria, seizures	1700 mg/kg weekly; unknown total length of treatment; report of 26 months for safety data	N/A	N/A	Pneumonia, sinus infection, rash with infusion	N/A
IV3	25 years	Schizophrenia type behavior, gaze palsy, dysarthria, dysphagia, hepatosplenomegaly, thrombocytopenia	2600 mg/kg weekly; unknown total length of treatment; report of 32 months for safety data	N/A	N/A	Tremors, chills, emesis, fever or headache during infusion (3 occasions)	N/A

SEQ1–9: patients received intravenous followed by addition of intrathecal treatment, IV1–3: patients received intravenous treatment only

*IV* intravenous, *IT* intrathecal, *IO* Intra-Ommaya, *VSGP* vertical supranuclear gaze palsy, *CVC* central venous catheter (Port-a-Cath), *N/A* not applicable

^a^LP: lumbar port placed for ease of administration and to eliminate sedation

A number of clinical assessment scales to assess severity and progression of disease in NPC have been utilized to monitor and measure clinical manifestations longitudinally over time, and in response to intervention [41, 43, 44, 67]. In 2010, a clinical severity score (adapted from prior clinical tools [67] was published by the National Institutes of Health (NIH) in conjunction with the NPC natural history study to assess progression of disease (primarily neurocognitive); and was validated for utilization in both a prospective and retrospective manner [46]. This tool utilizes nine major and 8 minor domains of clinical manifestations. These major domains include: gross motor, fine motor, hearing based on pure tone average, speech, cognition, memory, eye movements, seizures and swallow. The minor domains include: cataplexy, behavior, psychiatric symptoms, hyperreflexia, narcolepsy, continence, auditory brainstem response, and pneumonia. Each major domain is scored from 0 (no evidence) to 5 (severe manifestation); minor domains scored 0 to 2, and the maximum sum of the scores is 61. Higher scores correlate with more severe clinical signs and symptoms of NPC.

These tools provide practitioners a means for objective measurements and assessments of single clinical manifestations and do not require specialized testing (other than hearing). There can be a fair amount of inter-rater variability and the utility of these tools may rely heavily on methodology (written interpretation guidelines, videography) to limit this variability. All investigators reported in this paper utilized this scale to assess clinical status over time. For patients treated prior to the publication of this tool, the lead author (CH) reviewed medical records available and retrospectively calculated the scores for this publication and correlated scores for verification of data and consistency of reporting, in an effort to decrease inter-rater variability by adding another review. The majority of the scores were confirmed and only minor changes for interpatient consistency were adjusted in the final scores. Given the heterogeneity of clinical disease expression in this case series, as well as concerns for inter-rater variability, the specific assessments used in scoring were patient consistent so that each patient could serve as his/her own control.

All clinical protocols included safety assessments for monitoring of potential adverse reactions during and following drug administration based on the initial 2008 clinical protocol. Assessments included: periodic complete history and physical examinations, hearing evaluations (behavioral audiologic evaluations with calculations of pure tone averages at variable frequencies, tympanography, and, if clinically indicated, auditory brainstem response), laboratory studies (complete blood counts, chemistries, lipid panels, coagulation profiles, urinalyses), neurologic examinations (optional neuroimaging MRI and/or PET, EEG), neurocognitive and eye evaluations. The frequency of such testing was at the discretion of the investigator and institution as well as country or state review boards, though in most cases was temporally related to the treatments and intervals increased over time following months to years of safe administration. Adverse events (including severe adverse events, SAEs) were reported to local authorities and appropriate pharmaceutical companies, and the data also captured for this report.

## Results

### Demographics

The demographics, diagnostic studies and initial clinical presentation characteristics of the 12 patients included in this analysis are shown in Table 2. A narrative case history on each patient is provided in a Supplemental report (Additional file 1). Here we provide an overview of the patients, key disease features and details of treatment and outcomes with exposure to HPβCD.

**Table 2:** Patient characteristics and diagnostic studies, baseline

Patient identification	Gender	Age at diagnosis	Signs/Symptoms at diagnosis	Diagnostic tests NPC1 filipin/Genotype	Miglustat treatment
SEQ1	Female	3 years	Splenomegaly, pancytopenia, cognitive impairment	Cultured fibroblast, positive filipin; genotype heterozygous for c.1920delG exon 12 and IVS9 c.1554-1009G > A missense mutation	yes
SEQ2	Female	3 years	Splenomegaly, pancytopenia, cognitive impairment	Cultured fibroblast, positive filipin; genotype heterozygous for c.1920delG exon 12 and IVS9 c.1554-1009G > A missense mutation	yes
SEQ3	Female	7 years	Cognitive impairment	Cultured fibroblast, equivocal filipin; genotype heterozygous for c.1552C > T, p.R518W (p.ARG518Trp) in exon 9 and c.2594C > T, p.5865 L (p.Ser865Leu) in exon 17	yes
SEQ4	Female	5 years	Ataxia	Cultured fibroblast, equivocal filipin; genotype heterozygous for c.1552C > T, p.R518W (p.ARG518Trp) in exon 9 and c.2594C > T, p.5865 L (p.Ser865Leu) in exon 17	yes
SEQ5	Male	10 years	Dysarthria, cognitive impairment, anxiety, hypotonia, VSGP	Cultured fibroblast, positive filipin; genotype heterozygous for R978C missense and IVS21–2 A > G splice site mutation	yes
SEQ6	Female	7 years	Splenomegaly, VSGP	Cultured fibroblast, positive filipin; genotype heterozygous for R978C missense and IVS21–2 A > G splice site mutation	Yes
SEQ7	Male	2 years	Neonatal hepatosplenomegaly, conjugated hyperbilirubinemia, hypotonia, global developmental delay, failure to thrive	Cultured fibroblast, positive filipin; Genotype heterozygous for c.2008_2011delITGCT and c.3565_3566insG	yes
SEQ8	Female	1 year	Liver dysfunction, cholestatic jaundice, hepatosplenomegaly, developmental delay	Genotype heterozygous for c.2213C > A and c.3234_3237dupATTT	Yes
SEQ9	Female	20 years	Cognitive decline, ataxia, hepatosplenomegaly	Cultured fibroblast, positive filipin; Genotype heterozygous for c.688_69delTCTGTG and c.3182 T > C	No
IV1	Female	8 months	Hepatosplenomegaly, liver fibrosis, difficulty feeding	Cultured fibroblast, positive filipin; Genotype heterozygous for p.I923V and A1151T (c.2767A > G/3541G > A)	No
IV2	Male	15 years	Hepatosplenomegaly, behavioral disturbance, epilepsy, cognitive decline	LS-509 biomarker normal; Confirmed NPC1 gene mutation, results not available	yes
IV3	Female	16 years	Liver dysfunction at birth, hepatosplenomegaly, schizophrenia	LS-509 biomarker increased; genotype not available	yes

SEQ1–9: patients received intravenous followed by addition of intrathecal treatment

IV1–3: patients received intravenous treatment only

*VSGP* vertical supranuclear gaze palsy

We collected data from 8 investigators treating 12 patients with NPC. Nine of the 12 patients are female and three are male, and there are three sets of siblings including a pair of identical twins. Seven patients are Caucasian, 4 white Brazilian and 1 Asian. The mean age at diagnosis was 7.5 years with a range of 8 months to 20 years. The mean age of initiation of treatment was 13 years (range 21 months to 27 years) with a mean interval from diagnosis to treatment of approximately 5.5 years. The majority of patients (10 of 12) received miglustat prior to treatment and continued on the medication while receiving HPβCD. The data on length of treatment or patient compliance with miglustat are not available. At the time of intervention with HPβCD, miglustat was considered to be part of a standard of care regimen and the only published disease altering therapy.

Limited data are available for two patients (IV2 and IV3) with respect to duration of therapy (26 and 32 months, respectively) but are included here for contribution to the safety data. Both of these patients received Trappsol® Cyclo™ exclusively. Nine patients received Trappsol® Cyclo™, four exclusively (two for 83 months each). The five patients that transitioned HPβCD products (SEQ1, 2, 5, 6, 7) received Trappsol® Cyclo™ an average of 36 months, with a range of 11 to 52 months prior to the switch to the alternate HPβCD. Of the 9 patients receiving IV (including SEQ3 and SEQ4 exclusive use) the average duration of exposure to Trappsol® Cyclo™ was 44.8 months (range 11 to 83 months). In total, patients received HPβCD an average of 56.2 months (range 17 to 92). Patient exposure is therefore heavily weighted (80%) towards Trappsol® Cyclo™ in this group of patients. Patients received one or both HPβCD products but this report will not separate the clinical and safety outcomes by products and no data is available suggesting benefit of one over the other. For those patients who were exposed to both products, there was no change in status or adverse effects that could be attributed to change in formulation in careful review of the data.

Further details about the patients’ disease severity at initiation of treatment, treatment pathway (route, dose and interval), and adverse effects of treatment are provided in Table 1. All 12 patients received IV HPβCD. Nine of the 12 patients received IV treatment followed by the addition of IT sequentially (SEQ1–9). Three of the 12 patients received IV treatment exclusively (IV1–3). Decisions regarding IV and/or IT dosing, intervals and route of therapy were at the discretion of the investigator and patient family. For the ten patients with interval data, the duration of time patients received IV therapy ranged from 17 to 92 months and the average time of IV alone prior to IT for the sequential patients was 13 months (range 1 to 23 months).

The range of phenotypic expression of the disease (Table 2) ranged from relatively asymptomatic, juvenile onset (patient SEQ6 with a history of splenomegaly, mild VSGP and hyperreflexia) to severely affected early onset (IV1 and IV2, both with severe cognitive impairment, immobility, loss of language, and decreased ability to swallow and protect the airway). Nine patients had initial diagnostic testing utilizing cultured skin fibroblast for filipin staining (2 of whom had equivocal results) and 11 of 12 patients had genetic confirmation of *NPC1* and displayed an array of mutations as has been reported with this disease [11, 24, 68]. One patient’s diagnosis was based on results of the LS-509 testing and clinical features of the disease [69–71].

Most patients had surgical placement of central venous catheters to allow safer and more efficient delivery of the IV formulation. Nine patients received IT therapy and an additional 3 patients had Ommaya reservoirs placed for IO therapy. Two adolescent/young adult patients had lumbar ports place for ease of administration and avoidance of sedation (SEQ5 and SEQ9).

### Safety and adverse events

Adverse events are summarized in Table 1. SAEs requiring immediate reporting included a post-operative hemorrhage following placement of the Ommaya reservoir in patient SEQ2 as well as Port-a-Cath infections (SEQ3, SEQ4, IV1) and an Ommaya infection/meningitis (SEQ3). These SAEs were attributed to protocol devices and not to the drug. Two Port-a-Caths and two Ommaya reservoirs were removed due to these events; another Port-a-Cath required revision due to device malfunction. Two patients continue to have a long-standing Ommaya reservoir/catheter system (SEQ1, SEQ4).

The most common AEs were grade 1 and 2, requiring either no intervention or supportive measures alone, and included: infusion reactions with nausea (IV, IT) or headache (IT) and increased seizure activity for up to 24 h following IT (or IO) treatment. Increased seizure activity occurred in patients with a prior history of seizure activity (SEQ1, SEQ2, SEQ5, SEQ8). Patients SEQ3 and SEQ4 experienced transient worsening of ataxia, dysarthria and worsened fine motor control following high (1000 mg) intra-Ommaya (IO) doses which did not occur at lower (100–350 mg) doses. Patient SEQ5 experienced increased lethargy and ataxia for 1 week following IT administration at 600 mg, but no adverse events with a 500 mg dose. Patients also experienced periodic viral infections, otitis, sinusitis, diarrhea, and pneumonias not attributable to drug or disease. No patient experienced hearing loss as a result of IV therapy though two patients reported mild hearing loss in high frequencies with IT treatment (SEQ5, SEQ6). No patient discontinued the medication due to an adverse reaction.

A review of laboratory assessments (complete blood counts, chemistries, lipid panels, coagulation studies and urinalyses) do not show any trend or new abnormalities amongst the patients. Some patients had pre-treatment leukopenia, thrombocytopenia or anemia attributed to hypersplenism, which was noted to worsen transiently with viral infections (SEQ1, SEQ2). Most patients showed mild pre-treatment elevations of liver transaminases, with the exception of those with a history of cholestasis (SEQ7, SEQ8) in which the elevations were marked.

### Clinical severity scores and assessments

**Fig. 1:**
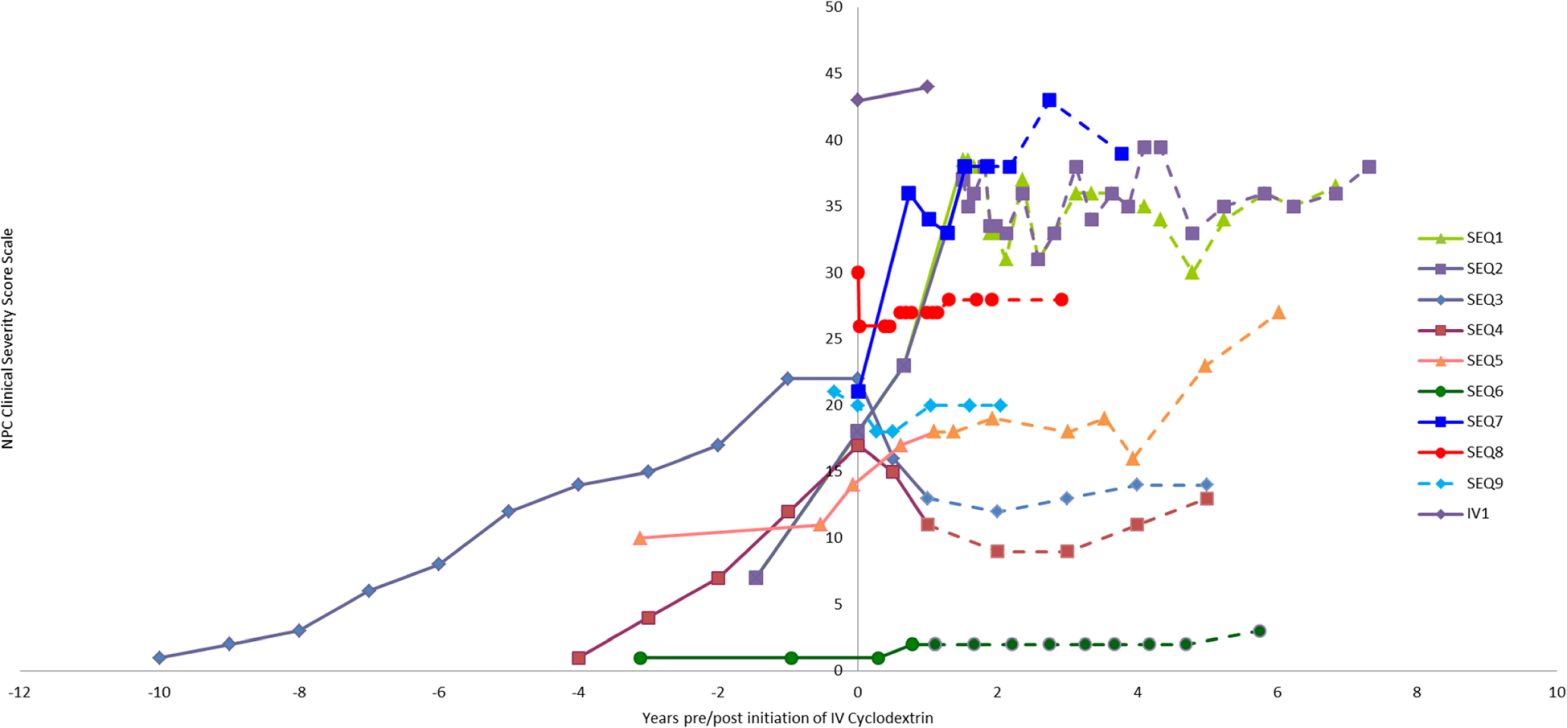
NPC Clinical Severity Scores: Pre- and post-IV infusion. The center vertical bar represents the time each patient initiated treatment with IV treatment. Scores obtained prior to treatment initiation are shown to the left of this bar. Solid lines to the right of treatment initiation represent IV only and dotted lines represent the addition of IT treatment for the sequentially treated patients

A number of patients had frequent assessments, but for consistency we have reported NPC Clinical Severity Scores (NCSS) at approximate 6-month intervals in this report (Fig. 1). The baseline scores prior to initiation of IV and IT are included, and, for patients with available medical records, retrospective scores have also been calculated to establish rate of disease progression. Each patient serves as their own control for comparison of severity scoring.

We performed a comparison of regression lines for patients with at least 3 data points (clinical severity scores) pre-and post-IV infusion of HPβCD with comparison of slopes and intercepts. *P*-values were calculated for overall test of coincidence. Table 3.

**Table 3:** Overall test of coincidence of regression lines

Patient ID	Slope pre-IV	Slope post-IV	*p*-value
SEQ1	7.55	0.96	< 0.001
SEQ2	7.55	0.72	< 0.001
SEQ3	2.32	−0.97	< 0.001
SEQ4	3.60	−0.75	0.015
SEQ5	1.00	1.39	0.616
SEQ6	0	0.18	0.236
SEQ7	N/A	4.19	n/a
SEQ8	N/A	0.37	n/a
SEQ9	N/A	1.29	n/a
IV1	N/A	1	n/a

Slopes were calculated independently for pre-intravenous (pre-IV) infusions and post-IV infusions, and when data was available for both, *p*-values were generated

*N/A* data not available, *n/a* not applicable

This is a descriptive study of compassionate use protocols (adapted for the individual patients in some circumstances) and therefore no power calculations were performed. The rate of change between the pre- and post-infusion time points, as calculated by the slope of the line between scores, proved statistically significant for patients SEQ1 and SEQ2, as well as patients SEQ3 and SEQ4. The *p*-values did not suggest relevance for patients SEQ5 and SEQ6. These values were calculated as related to time of initiation of IV infusion. Though nine patients sequentially received IT therapy there were not enough time points to create 2 sets of post-treatment lines (with calculated slopes) to determine if statistically significant changes occurred with the sequential addition of IT therapy. Observation of the slopes in Fig. 1 suggests the patients did not show added benefit (as measured by a decrease in the clinical severity score) when IT (or IO) therapy was added to the treatment regimen. How CNS directed therapy (in SEQ patients) contributed to disease stability is not possible to determine.

Patients presenting with severe clinical manifestations (typically with NCSS over 30) demonstrate progression of disease with increasing scores over time and then appear to plateau (no change in scores). Patients with an apparent plateau may still show periodic declines but not to a degree that will alter the NCSS, and this adds another element of complexity in the interpretation of results. It is unknown if our findings may represent natural history of disease progression or is an effect of intervention. Two less severely affected patients had immediate and notable decreases in their scores (SEQ3, SEQ4) with initiation of IV therapy, and though some progression was seen years later, these patients never reached the pre-infusion level of clinical severity scores. The change in scores, as well as the reports from the investigators and patient families (not all observed changes are quantified with the NCSS), indicate improvement in clinical symptoms of disease. Of interest, patient SEQ6 presented relatively asymptomatic and has remained so for the duration of the treatment. Given the patient’s stability, it is not possible to determine the natural history for this patient versus a changed course due to this intervention.

**Fig. 2:**
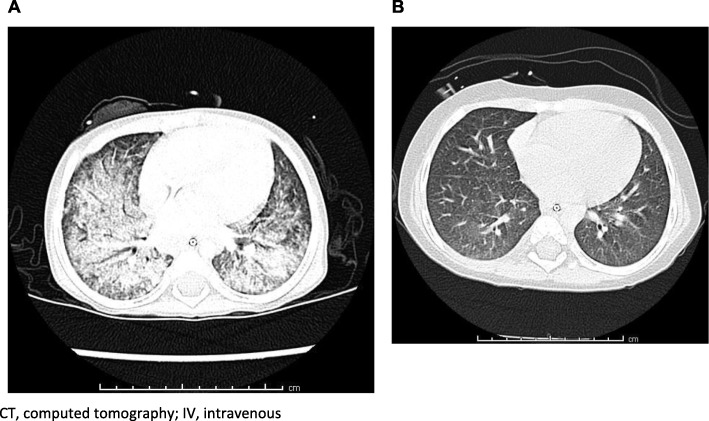
CT scans for Patient SEQ7 prior to (**a**) and after 9 months of treatment (**b**) with IV HPβCD. CT, computed tomography; IV, intravenous

A surprising finding was improvement in some of the neurologic (fine and gross motor, swallowing), neurocognitive and/or behavioral and psychiatric manifestations of the disease in two patients (SEQ3 and SEQ4). Additionally, one patient with lung disease (SEQ7) (Fig. 2 CT scans for Patient SEQ7 prior to (A) and after 9 months of treatment (B) with IV HPβCD) and another with massive hepatomegaly and elevated AST (SEQ8) (Table 4) showed clinical improvements related in time to initiation of IV (and prior to IT) treatment. The beneficial effect of HPβCD on lung disease in one of our patients (SEQ7) is an interesting observation considering that the results of in vivo studies showed that treatment with HPβCD had a minimal effect on lung cholesterol and collagen levels in an NPC mouse model and caused pulmonary toxicity in an NPC cat model [60, 73]. The differences in these results may be due to influence of parenchymal disease or the production of specific lung secretions, or be species specific.

**Table 4:** Change in liver volume and liver enzymes in patient SEQ8

Age at Liver MRI	Treatment IV, IT HPβCD	Liver volume (cm^3^)	Liver volume/ body weight (cm^3^/kg)	Normal range liver volume/body weight (cm^3^/kg) [72]	AST (U/L) RR 15–46	ALT (U/L) RR 3–35	GGT (U/L) RR 5–55	AP (U/L) RR 80–270
1 year 9 months, Baseline	Pre-IV	772	91.4	28.56 +/−5.4 (1 year, 6 months)	88	37	116	274
2 years 2 months	Continued IV, pre-IT	767	71.1	31.83 +/− 5.9 (3 years 4 months)	75	28	102	189
2 years 9 months	IV/IT	699	58.5	31.83 +/− 5.9 (3 years 4 months)	41	28	77	171
3 years 9 months	IV/IT	777	49.8	31.83 +/− 5.9 (3 years 4 months)	40	36	< 10	159
4 years 11 months	IV/IT	812	47.5	31.83 +/− 5.9 (3 years 4 months)	41	37	113	169

Absolute volumetric liver measurements are shown with respect to age and timing of either IV, or sequential IV + IT treatment. A marked decrease in liver size relative to weight and expected volume by age [72] is seen following initiation of IV treatment and continues to decrease with sequential treatment. Liver enzymes show variability at baseline though AST decreases from twice upper limit of normal to a normal range to normal following 1 year of treatment

*MRI* magnetic resonance imaging, *IV* intravenous, *IT* intrathecal, *AST* aspartate aminotransferase, *ALT* alanine aminotransferase, *GGT* gamma-glutamyl transpeptidase, *AP* alkaline phosphatase, *U/L* units per liter, *RR* reference range

Patients less severely affected appeared to benefit more with improvements or stability than those more severely affected, and not all these improvements related to quality of life were captured in the NCSS. Another interesting observation was that the addition of IT therapy (or IO) did not result in additional improvement in symptoms.

Review of the medical records note a number of patients experienced increased well-being attributable to increased ability to focus or increased alertness resulting in improved communication, less confusion, improved behavior and better ability to manage activities of daily living. These changes were reported by the parents as well as treating physicians for patients SEQ1, SEQ2, SEQ3, SEQ4, SEQ5, SEQ6, SEQ7 and SEQ9. As this was not part of the clinical severity score or protocol, this information on general well-being or quality of life (utilizing validated tools) was not sought and is anecdotal, but we note these comments in the medical records here to be of interest. Formal quality of life assessments should be addressed in future clinical trials.

## Discussion

In this case study analysis, we report on the safety and clinical observations following the administration of HPβCD in 12 patients with NPC (11 genetically confirmed *NPC1*). These cases include patients who have received long durations of IV treatment with HPβCD (range 17 to 92 months) and highlight important safety and efficacy data following long-term use. We did not encounter any serious adverse event (SAE) in the administration of IV HPβCD. Some patients experienced mild infusion reactions, which were subsequently prevented with use of anti-emetic pre-medication or assuring adequate hydration. The patients were able to transition to receive home IV infusions given the ease and safety of administration.

### Impact of intravenous HPβCD on clinical course

The efficacy data arising from our case series suggest clinical improvements in the systemic and neurologic manifestations of NPC disease following the IV administration of HPβCD. It is emphasized that this is a case series and not a powered study, confounded by patient heterogeneity and variability in interventions. We report here our observations suggesting a clear safety profile as well as potential benefit, that support the need for formal clinical trials. The observed improvements include reduced hepatomegaly, improved transaminase levels, improved fine and gross motor control, improved behavior with amelioration of psychiatric symptoms, and the resolution of interstitial lung disease (in one affected patient). The improvements in measurable neurocognitive function in some patients, as well as the unsolicited reports of increased alertness and focus in even the most severely affected patients, conflict with our prior notion that HPβCD does not cross the blood brain barrier (BBB) and would therefore not be expected to affect clinically apparent change [74]. However, we cannot exclude the possibility that HPβCD is crossing in low concentrations sufficient for response or is acting at the level of the BBB regulating brain cholesterol metabolism, by means of signaling or changes in paracellular permeability or tight intercellular junctions, altering membrane structures, or by influencing solubilization of lipids of brain endothelial cells [75–79]. Cyclodextrins directly interfere with biological membranes, without penetration, to extract lipids such as cholesterol and are known to influence cell metabolism and function [76].

Interestingly, many of the patient families and healthcare providers in this case series anecdotally reported observations including an increase in level of patient alertness, ability to communicate and general well-being leading to improved quality of life following initiation of IV infusions, clinical assessments not captured in the NCSS tool. These changes would indicate that additional measures in quality of life would be beneficial to include in future trials. Additionally, measures of change in systemic manifestations of disease need to be developed to address a sub-population of patients, as these areas are not specifically measured in the NCSS.

The benefits observed in these cases were reported early in the patients’ treatment pathway (often within several doses) and were sustained throughout the duration of the IV HPβCD treatment. It would not be possible to determine an added effect of each agent (including prior miglustat treatment) in the long-term disease progression, though a number of non-neurologic as well as immediate changes noted with initiation of HPβCD suggests a true benefit of this latter agent. Given that miglustat is currently the only published disease altering treatment, future clinical trials with other agents will need to address the same issue.

Furthermore, we did not observe new or immediate changes or improvements amongst the cases in which IT therapy was subsequently added to IV treatment. Of note, 75% of the patients added IT administration to IV administration within 1–2 years of initiating IV therapy. Patients who were less severely affected at initiation of IV treatment experienced more benefit than those with severe manifestations, and likely this is due to the degree of irreversible damage present in some patients. A number of the patients in this case series were severely compromised at the time of treatment initiation and indeed this may have precluded the possibility of clinical benefit. Prior preclinical studies in mouse and feline NPC models demonstrated that young animals responded more favorably and older animals showed lesser benefits, again supporting the notion that age and severity are important factors with respect to response (or possible benefit) to treatment [53, 55, 60, 80]. The majority of patients remained clinically stable and no additional improvements were noted with the addition of IT therapy (with the exception of the identical twin patients SEQ1 and SEQ2 who experienced an improvement in hearing on a stable low dose meeting certain concentration goals as measured on pharmacokinetic sampling and modeling). It is not possible to determine if disease stability or slowing of progression is related to IV and CNS directed therapy, or an additive effect of miglustat and HPβCD. Improvements in systemic manifestations (liver, lung) could not be explained by miglustat treatment.

### Safety assessment and adverse events

The safety data obtained in this analysis showed that several AEs were reported following the administration of HPβCD. However, some of these adverse events were consistent with the natural history of NPC. In total, we believe that two notable safety findings were reported in these case studies, namely systemic reactions to the IV administration of HPβCD and AEs associated with implanted devices designed to facilitate long-term parenteral or CNS directed administration. The increased seizure activity observed in some patients receiving IT HPβCD (with a known prior history of seizure activity) is presumably due to a transiently lowered seizure threshold, though the mechanism is unknown. Data was not collected to specifically evaluate this relationship with dosing, frequency, route (IO/IT) or other factors which may affect neurologic irritability. Additionally, as measured by behavioral audiologic and ABR testing, high frequency hearing loss was reported in some patients following IT therapy, as has been previously reported in the NIH clinical trial, but not in the patients receiving IV therapy [81]. The systemic reactions that were noted did not lead to treatment discontinuation and are not unusual with IV infusion of a therapeutic agent. These reactions were readily managed with conventional clinical practice and specific measures could be initiated to limit their occurrence in the future. Given the progressive, degenerative nature of NPC, it may be difficult to distinguish safety related treatment adverse events from those related to disease progression. Nevertheless, based on the safety data we report here, we consider that no unexpected safety issues were experienced.

Initial case reports have been previously published (utilizing the Hastings/Hempel protocol) of the use of HPβCD to treat patients with NPC. Improvements in hepatosplenomegaly and CNS dysfunction were observed following the administration of IV HPβCD to two Japanese patients with NPC [82]. However, no improvements in neurological deficits were observed in that report. Of note, these patients appeared to have been severely neuro-cognitively impaired and such damage was likely already irreversible. The investigators subsequently reported on IT administration of HPβCD in an 8 year old girl with perinatal onset of disease, who remained stable with severe neurologic dysfunction for 2 years [83]. Of note, the authors state the parents observed the patient to become more alert. Furthermore, no adverse effects were observed over the course of treatment. In a subsequent case report, IV HPβCD was administered twice weekly, following the Hastings/Hempel protocol, to a single patient at a dose of 2500 mg/kg over 8 h to a 4 year old girl with hepatosplenomegaly and neurocognitive decline [59]. Pharmacokinetic studies were done and comparable effective drug concentrations were achieved which were similar to the in vivo studies in mice reported by Liu et al. [55, 59]. The authors did not report on clinical outcomes but noted no adverse effects. Two additional case reports of IT HPβCD in patients have recently been published [84, 85]. Two adult late onset patients received IT HPβCD without improvement or change in progression, and the authors attribute this lack of improvement to be related to age and severity of disease [84]. A recent review of published cases of HPβCD treatment for NPC summarized 17 patients, including abstracted data from some of the patients reported in this case study, note the significant toxicities (hearing loss, meningitis) were seen in patients receiving CNS directed drug [86]. Results were suggestive that efficacy may be partial and dependent on multiple factors including severity of disease, timing of drug initiation with respect to disease progression, route and dose of HPβCD, and other interpersonal variables.

An emerging area of interest and investigation is the association of NPC with gastrointestinal (GI) symptoms. A recent association of NPC with Crohn’s phenotype has been reported [87] as well as ineffective carbohydrate metabolism [88]. These reports point to evidence of systemic disease in some affected individuals with NPC that may experience particular benefit with treatment aimed at affected peripheral organs. A recent study examining impact of a small molecule, ursodeoxycholic acid (UDCA) believed to act by rescuing a suppressed cytochrome P450 system, on liver function in NPC patients showed stabilization or improvement of liver enzymes [34]. This drug was delivered systemically, well tolerated, and appeared to have neurologic benefit based on improvement in clinical severity scores and parent reported neurological benefits, including increased alertness, improved sleep patterns, and increase in appetite. More investigation is needed in this area.

### Limitations

This descriptive review of compassionate use data in 12 patients does have limitations by virtue of its nature. Our review is a descriptive analysis and was not designed as a clinical study. We have also provided a historical account of the investigators’ process of bringing HPβCD as a potential intervention to patients with NPC. As discussed, we performed a retrospective review of cases, though some of the data was collected prospectively. Some of the clinical NCSS scoring was done post-hoc for patients treated prior to this published tool in 2010 and drew upon a detailed review of the medical records. The patients were not standardized by degree of disease severity, age, length of symptoms, or type of clinical manifestations, as might be controlled in a clinical trial. Patients serving as their own controls in natural history as well as clinical trials in rare disease with such variability of genotypic/phenotypic expression is a means to address some of these issues. Some symptom assessments were subjectively recalled by caregivers potentially biased by an open-label placebo effect. A confounding factor of this case series is that the population of patients is very heterogeneous in terms of age, disease severity, length of symptoms prior to treatment, rate of disease progression, and route (and dosing) of HPβCD administration. Many patients received supplements and other medications (including miglustat) to address underlying consequences of NPC such as seizures, and this could not be controlled for this case series. The data acquired therefore need to be considered on an individual as well as on a collective basis.

The NCSS, a validated tool for clinical assessment in NPC, has limitations in that not all clinical signs or symptoms of NPC are captured. This tool primarily utilizes assessments of neurocognitive and not systemic disease manifestations [46]. Additionally, it may not be reasonable to assume all patients would follow the same trajectory of disease progression, and indeed some patients may seemingly plateau at certain levels though may still have periods of continued decline at variable rates and perceptibility. As well, not all patients may be expected to continue to progress in or reach the most extreme outcome in each domain. It was also noted that in one patient (SEQ5) a tonsillectomy led to notable clinical improvements in swallow and speech, the severity of which had been previously attributed to NPC. Not all changes on the NCSS will be a direct result of disease progression or improvement related to effective treatment. The NCSS was validated in patients ages 4 to 51 years of age, and these assessments are indeed difficult to ascertain in the child younger than 4 years, and the progression may not be so predictable when accounting for variability in developmental milestones. A recently developed tool, denoted annual severity increment score (ASIS), addressed the variability in progression of disease as related to age [89]. This score has promise to serve as a prognostic tool for new treatment interventions.

In this report, we have attempted to determine if expanded access IV HPβCD intervened with the projected natural history of disease progression as predicted by the NCSS tool. We applied linear regression modeling for individual patients, whereas the previously reported validation of the NCSS tool averaged the slopes for all patients. Our mathematical modeling suggests that for some of the patients the change in slopes pre-and post-infusion were not coincidental and indeed likely represent an effect of intervention. Our analyzable data is limited by the number of data points available for all patients for both pre- and post-infusion, and true calculations for significance could only be done for those with an adequate number of data points. We compared the trajectory of progression for individual patients based on their pre-infusion rate of change (each patient served as his/her own control). Based on the previously reported NCSS validation study, once patients become symptomatic it is anticipated they will continue to have progressive disease as measured by an increasing score over time [46]. However, we could not know with certainty that each patient would indeed follow a specific rate of progression given the variability in severity of disease in our patient population.

Outcome for one of our cases (SEQ6) suggests that IV HPβCD, when provided to an asymptomatic patient, could have a preventative effect in symptom development. Intervention with HPβCD in asymptomatic animals (mice and cats) prolonged life as well as led to marked delays in disease progression [53, 55, 60, 80]. Based on this patient’s low and stable NCSS for the duration of treatment (68 months) we cannot predict with certainty what the disease trajectory would have otherwise been in this patient. The patient’s course has differed from that of the sibling, who was initiated on therapy after already developing manifestations of the disease, though it is known siblings may vary in their clinical course [1, 90–92]. The patient’s clinical stability is nevertheless intriguing and brings up the question of the importance of timing to initiate a potentially effective intervention.

We captured some clinical changes (albeit anecdotally) that impacted quality of life in a positive manner following IV treatment in the medical records and with discussion between patients, families and physicians. Given this intriguing finding, it will be critical to include Quality of Life assessments for future clinical trials that will assess such measures as alertness, ability to focus (improved ability to read), specific behavior changes, school and family relationships.

## Conclusions

These case studies demonstrate that IV HPβCD is a well-tolerated treatment that can potentially treat systemic and neurologic manifestations of disease in patients with NPC1. While progressive decline was expected in accordance with previously published natural history data [24, 46], the rate of progression appears to be less than expected in some individuals receiving IV HPβCD. Optimal dosing, dosing interval and route that will enhance outcome for patients with NPC are yet to be determined.

A multi-system disease such as NPC requires a multi-targeted treatment approach. It is also important to consider the heterogeneity of the disease as an approach in one patient may not be the most appropriate for another. HPβCD will certainly play a significant role in targeting the cellular cholesterol burden known to be present in NPC, a mechanism different from that of miglustat which targets sphingosine and glycosphingolipids. Novel agents are urgently needed.

Our series of patients presented here represent the largest collection of patients treated with IV HPβCD. With demonstration of a favorable safety profile and encouraging clinical outcomes, further validation with a randomized clinical trial is needed. Two formal clinical trials are currently underway to evaluate safety, pharmacokinetic and pharmacodynamic assessments of systemic IV administration of HPβCD.

## Supplementary information

**Additional file 1.** Supplemental Data: Case Summaries on Compassionate Use HPβCD to accompany the manuscript “Expanded access with intravenous hydroxypropyl-β-cyclodextrin to treat children and young adults with Niemann-Pick disease Type C1: A case report analysis”. Narratives.

## Data Availability

The data that support the findings of this study are available on reasonable request from the corresponding author and/or CTD Dr. Sharon Hrynkow. However, restrictions apply to the availability and use of these data, which were specifically requested for use in this review of compassionate use, and so are not publicly available. Access to the initial Hastings/Hempel clinical protocol (2008) for compassionate use, and FDA filings, can be viewed online [57].

## Abbreviations

AE: Adverse event
BBB: Blood brain barrier
CNS: Central nervous system
CRF: Case report form
FDA: Food and Drug Administration
GI: Gastrointestinal
HPβCD: Hydroxypropyl-β-cyclodextrin
IND: Individual New Drug
IO: Intra-Ommaya
IRB: Institutional Review Board
IT: Intrathecal
IV: Intravenous
NCSS: NIH Clinical Severity Scale
NIH: National Institute of Health
NPC: Niemann-Pick disease Type C
SAE: Serious adverse event
SEQ: Sequential, patients treated IV then added IT
VSGP: Vertical supranuclear gaze palsy
