# Supplementary material for: Expanded access with intravenous hydroxypropyl-β-cyclodextrin to treat children and young adults with Niemann-Pick disease type C1: a case report analysis
Source: Orphanet J Rare Dis. 2019 Oct 21;14:228. doi: 10.1186/s13023-019-1207-1 (PMC6805667; doi:10.1186/s13023-019-1207-1)
Supplement: Supplementary file 1 — Additional file 1. Supplemental Data: Case Summaries on Compassionate Use HPβCD to accompany the manuscript “Expanded access with intravenous hydroxypropyl-β-cyclodextrin to treat children and young adults with Niemann-Pick disease Type C1: A case report analysis”. Narratives. [file 13023_2019_1207_MOESM1_ESM.docx]

1. **Supplementary Data**

A narrative case history on each patient is provided in this section.

## IV followed by concurrent IV and IT treatment

Nine individuals (SEQ1 to SEQ9) received IV followed by concurrent IV and IT treatment with Trappsol® Cyclo™ and are referred to as sequential (SEQ) patients.

Patients **SEQ1** and **SEQ2** are Caucasian female identical twins who were diagnosed with *NPC1* at 3 years and 9 months of age by fibroblast culture technique (abnormal filipin staining), confirmed with genetic mutation analysis. The girls presented at 16 months of age with splenomegaly and mild pancytopenia, with subsequent severe developmental delay apparent by 2 ½ years of age. The patients initiated treatment with miglustat one month following the diagnosis of NPC. These patients were the first to receive compassionate use Tappsol® Cyclo™ and initiated systemic therapy in April 2009, at the age of 5 years, 3 months. Collaboration with the FDA led to the development of a dose escalation intravenous protocol based on safety outcomes. From diagnosis to initiation of treatment (a lapse of 18 months), the patients progressed with more significant ataxia and need for assisted ambulation, loss of new language acquisition, development of vertical supranuclear gaze palsy (VSGP) and dysphagia. The patients received IV doses initially at 80mg/kg with escalation to 2800 mg/kg Trappsol® Cyclo™ twice weekly. Assessments for safety and toxicity included: laboratory evaluation with complete blood counts, complete metabolic panels, lipid panels, urinalyses with protein to creatinine ratios, and basic coagulation tests. Imaging consisted of volumetric computed tomography (CT) for liver and spleen size, brain positron emission tomography (PET) imaging as previously reported,[55] and chest CT with bronchoscopy and pulmonary function tests (PFTs). Bronchoscopy performed within the first few months of IV therapy demonstrated xanthomas in the airways of both children, which were no longer visible one year later. No serious adverse effects were seen, and the maximal IV dose administered was 2800 mg/kg twice weekly. Interestingly, the blood counts improved despite persistent splenomegaly and resultant hypersplenism, though would worsen acutely with viral illnesses. To date, the patients continue to receive 2500 mg/kg as a weekly 8 hour IV infusion. Pharmacokinetic studies were done at the 2500 mg/kg dose (data not shown) and confirmed the plasma concentration target concentration of between 1-3 mM (see discussion).

Eighteen months following initiation of intravenous HPβCD, the patients began sequential therapy with intrathecal (IT) doses of initially 175mg, then 350 mg Trappsol® Cyclo™ every 2 weeks. Two and one half years later, both patients had an Ommaya reservoir implanted to facilitate directed treatment into the central nervous system (CNS). Pharmacokinetic studies were performed and determined a dose of 50 mg IO (intra-Ommaya) achieved similar concentration of drug in the cerebrospinal fluid as 350 mg delivered IT without reaching potentially neurotoxic concentrations(data not shown).

Following a post-operative complication of cerebral hemorrhage in patient SEQ2, the reservoir was removed 1 week after insertion. The hemorrhage was thought to be a rare unfortunate complication of the procedure. A detailed bleeding assessment was performed without any evidence of an inherited or acquired bleeding disorder that would increase risk. Transient clinical deterioration as measured by increased clinical severity scores was noted in this patient post hemorrhage.

Clinically relevant improvements in certain parameters were noted for both patients during IV therapy alone. These included sustained improvements in swallowing, ataxia, and enhanced human interaction and level of alertness. Sequential dosing with IV and IT led to continued stabilization for several years and slower than anticipated disease progression, though no additional improvements (other than improved hearing were noted as a result of the addition of low dose IT therapy. Disease progression was also reported for both patients following sequential therapy, with difficult to control seizures and cataplexy episodes, both attributed to disease progression. Both patients experienced SAEs of increased seizure activity in the 24 hours following IT treatment and Patient SEQ2 also experienced an SAE requiring removal of the Ommaya reservoir due to post-operative delayed parenchymal hemorrhage. As IV treatment did not present any safety concerns, HPβCD treatment was considered sufficiently well tolerated to permit home-based therapy. Approximately 4.5 years after initiation of treatment, an alternative HPβCD (Kleptose®) was substituted for Trappsol® Cyclo™. The patients continue on IV infusions weekly and IT every 2 weeks and have been noted to have continued, albeit slow, global disease progression (non-ambulatory, swallow limited to solid foods) with preservation of hearing and better controlled seizures.

Patient **SEQ3** is a Brazilian female who was diagnosed with *NPC1* when she was 13 years old, after presenting with cognitive delay at age 7 years. Her diagnosis was based on genetic analysis following equivocal results of the filipin staining of cultured skin fibroblasts. She was initiated on miglustat at age 14 years. Prior to compassionate use IV Trappsol® Cyclo™ at age 15 years, the patient’s cognitive decline had significantly progressed and she also developed fine and gross motor difficulties, incontinence, and VSGP. Most significant were the worsening of her behavior as well as development of severe psychosis leading to difficulty in attending school and interacting with family and friends. She also had mild leukopenia without infections. Trappsol® Cyclo™ treatment was commenced at an initial dose of 1200 mg/kg twice weekly, and continues at 2500 mg/kg weekly. Sixteen months after initiating IV treatment, IT therapy with Trappsol® Cyclo™ was started at a dose of 175 mg every 15 days, with dose escalation to 350 mg and subsequent placement of an Ommaya reservoir for directed therapy (currently at 100 mg every 15 days). Treatment with Trappsol® Cyclo™ has continued without adverse events and the patient receives home IV infusion. The patient experienced an initial improvement in her condition following IV treatment with significant improvements in her behavior, increased legibility of her handwriting, and overall improved general well-being. No other interventions occurred during this time to account for these changes. She was able to return to school, re-engage in her social life and complete her high school education. Additionally, her self-esteem improved as did her quality of life (and that of her family) due to the positive effects on behavior and psychosis. The parents report not previously being able to travel with the children (SEQ3 and SEQ4) due to behavior problems and lack of control, including outbursts at mealtimes, but after IV therapy the family was able to resume travel. She has maintained a stable course on sequential therapy with IV therapy 6 years 11 months ago. The patient experienced two SAEs of Port-a-Cath infection (two episodes without identified pathogens) with removal of the device after the second infection. Of note, increases in IO dosing (350 mg) lead to transient worsening of her neurologic symptoms (ataxia, speech) which reverted to baseline with decrease in IO (now IT) dosing. No additional improvements were noted with sequential addition of IT or IO therapy. Following successful management of the SAEs, no further medical events of concern have been reported.

Patient **SEQ4** is a Brazilian female who was diagnosed with *NPC1* at 9 years of age and is the sister of Patient SEQ3. Her initial symptom at 5 years of age was difficulty with ambulation (ataxia). Her diagnosis was made on genetic analysis as her cultured skin fibroblast filipin staining was equivocal. She initiated miglustat therapy at age 10 years, and at the time of initiation of IV Trappsol® Cyclo™ infusions at age 11 years, she had developed severe cataplexy, moderate VSGP, as well as worsening cognition and memory, a gait abnormality and seizures. She was noted to have mild leukopenia without infections. IV treatment was commenced at an initial dose of 1200 mg/kg Trappsol® Cyclo™ twice weekly and continues at 2500 mg/kg weekly. IT therapy with Trappsol® Cyclo™ was added sequentially 16 months after initiation of IV therapy at an initial dose of 175 mg every 15 days, with dose escalation and subsequent placement of an Ommaya reservoir for directed therapy. Treatment with Trappsol® Cyclo™ has therefore continued without adverse effects and the patient receives home IV infusion. Treatment with Trappsol® Cyclo™ was associated with an initial improvement in her condition following IV administration that included marked improvements in behavior and fine and gross motor coordination, allowing her to return and complete high school and re-engage in social activities. She has had a stable course thereafter, and at the time of this report has received IV therapy for 6 years 11 months. The family reports a significant improvement in quality of life following IV therapy with improvements in behavior, psychiatric symptoms, self-esteem and general well-being, as well as decreased ataxia and improved fine motor coordination. The patient experienced two SAEs with Port-a-Cath infections (pathogens not identified), necessitating removal following the second infection.

Patient **SEQ5** is a Caucasian male who was diagnosed with *NPC1* at 10 years of age. He had been noted to have jaundice as a newborn and mild splenomegaly at 4 years of age. The patient presented at age 7 years with heading thrusting, hypotonia, and ataxia and his symptoms progressed to frank VSGP, cerebellar ataxia, dystonia and increased problems with math as well as social immaturity. A diagnosis of NPC was achieved by positive filipin staining on skin fibroblast culture and confirmatory genetic testing. Between diagnosis and initiation of IV infusions of Trappsol® Cyclo™ at age 13 years, his symptoms progressed and he developed dysarthria, dysphagia, cataplexy and partial complex seizures. He had also been diagnosed with attention deficit disorder. He started treatment with miglustat at 10 years of age and anticonvulsants to treat the seizures. The patient first received IV treatment at a dose of 2000 mg/kg Trappsol® Cyclo™ twice weekly. Clinical improvements were noted by the family and healthcare providers, as well as the patient, and included increased ability to focus and read, improved swallowing, lessened anxiety, and a halt in progressive decline with respect to school functioning (math and reading predominantly). His family noted him to be more physically active, have more energy and be more socially engaged. Additionally, weight control had been problematic with continued weight loss, but he began to eat better and gained weight. IT therapy with 350 mg Trappsol® Cyclo™ every 2 weeks commenced 13 months later. The patient experienced increased seizure activity in the first month, which led to alterations in anti-convulsant medication. A lumbar port was placed to facilitate the procedure and eliminate the need for sedation. Escalation of the IT dose to 600 mg resulted in high frequency hearing loss and the dose was subsequently reduced to 500 mg IT every 2 weeks. Change in formulation to an alternate HPβCD (Kleptose®) occurred at 2 years and 10 months from start of treatment. The patient was able to successfully transition IV therapy to home infusion. Of note, the patient underwent surgical re-construction for mandibular hypoplasia at age 18 years with notable improvement in chewing and swallowing. The use of IV Trappsol® Cyclo™ was not associated with any clinically significant AEs. He does experience increased lethargy and ataxia for approximately one week following IT administration. The patient does continue to show very slow progression though is able to perform activities of daily living, ambulate without assistance, speak and eat well.

Patient **SEQ6** is a Caucasian female who was diagnosed with *NPC1* at 7 years of age and is the sister of SEQ5. Medical history is remarkable for moderate splenomegaly (discovered at 3 months of age) and mild VSGP and hyperreflexia noted only on examination by a physician. She had no neonatal jaundice like her brother SEQ5 and no neurologic or cognitive deficits and no learning disability. She continues in school and is an excellent student and is physically active. NPC diagnosis was obtained by means of filipin staining of cultured skin fibroblast and confirmatory genetic testing. Miglustat treatment was initiated following confirmation of diagnosis. She was diagnosed with precocious puberty at 9 years of age, and it is unknown if there is any association with the diagnosis of NPC. Patient SEQ6 commenced IV treatment at a dose of 2000 mg/kg HPβCD at age 10 years, with very mild clinical stigmata of the disease (no progression noted since diagnosis). The patient received Trappsol® Cyclo™ for the first 2 years 6 months, followed by Kleptose®. Similar to her brother, Patient SEQ6 initiated IT therapy at a dose of 350 mg HPβCD every 2 weeks starting 10 months from initiation of IV therapy. The IT dose was subsequently increased to 500 mg HPβCD every 2 weeks after just over 3 years at 350 mg, and has developed high frequency hearing loss with the increased dose. Although no substantial improvements in the neurological condition of the patient were observed since she started HPβCD therapy, none of the declines that are usually associated with this progressive disorder have been observed either. The patient was able to successfully transition IV therapy to home infusion. The patient has not experienced any significant adverse effects since starting HPβCD therapy.

Patient **SEQ07** is a Caucasian male who was diagnosed with *NPC1* at 2 years of age. The patient’s medical history included neonatal hepatosplenomegaly, unconjugated hyperbilirubinemia, followed by early onset global developmental delay, failure to thrive, and VSGP as noted by the mother. A liver biopsy was inconclusive and he was treated for presumptive giant cell hepatitis without improvement. Diagnosis of NPC was achieved by cultured skin fibroblast filipin staining and confirmatory genetic testing. At 2 years 2 months of age, he was the youngest at the time to receive Trappsol® Cyclo™ IV at doses between 1500 and 2000 mg/kg weekly. He initiated miglustat treatment one month prior to the IV infusions of Trappsol® Cyclo™. His family and health care providers noted increased alertness, more verbalizations, increased energy and more interest in taking liquids within a month of IV treatment. He also appeared to be better able to handle respiratory secretions. The patient also had restrictive interstitial lung disease and required biPAP. Three months after starting IV treatments, a clinical hold for compassionate use HPβCD was placed and lasted 6 months, during which time the patient experienced acute worsening respiratory distress, hypoxemia with tachypnea, and loss of the ability to sit as well as all language skills. Following resumption of Trappsol® Cyclo™ treatment, the patient experienced significant improvement in his respiratory status and multiple repeat chest computed tomography (CT) scans showed dramatic improvement in his interstitial lung disease. See **Figure 1.**  Furthermore, treatment re-initiation was also associated with a recovery of some of his verbal skills. The patient subsequently started IT therapy (with Kleptose®) 23 months after start of IV, and he received doses of between 150 and 750 mg HPβCD every 2 weeks. IV HPβCD was changed at this time as well to Kleptose®. During his treatment with IV HPβCD, the patient experienced a variety of AEs including several episodes of acute on chronic respiratory failure, which required additional hospital admissions and was felt to be due to worsening neurologic status and progression of underlying disease. All other AEs were consistent with the natural history of NPC. Overall, early treatment with Trappsol® Cyclo™ appeared to stabilize disease progression at an acceptable rate of adverse effects, with periods of progression most notable during the 6 month clinical hold, as well in the last few months of his life. Treatment was discontinued by his healthcare providers and the patient died at the end of the clinical reporting period for this publication.

Patient **SEQ8** is an Asian female fraternal twin, who was diagnosed with *NPC1* just prior to 1 year of age. She was a 33 week premature infant and at birth was noted to have marked hepatosplenomegaly, thrombocytopenia, and cholestatic jaundice. She also has a history of dysphagia requiring gastrostomy tube placement and tracheobronchomalacia, requiring tracheostomy and ventilation, not thought to be due to NPC. Diagnosis of *NPC1* was made on whole exome sequencing. Beginning at 7 months of age, the patient experienced multiple episodes of left eye deviation, arm flexing and was treated with anti-convulsant medications for 8 months, though without definitive proof of seizure activity based on normal video electroencephalogram. Prior to the start of HPβCD she was noted to have persistent marked hepatosplenomegaly, and underwent a complete splenectomy for torsion and ischemia. She was non-verbal and non-ambulatory, making cognition difficult to ascertain. Eight months following her diagnosis, and at 21 months of age, the patient initiated IV HPβCD (Kleptose®) 500 mg/kg weekly, escalating to 2000 mg/kg weekly. Two weeks following initiation of IV HPβCD she developed status epilepticus and was re-started on levetiracetam. IT HPβCD treatment was started 5 months later at an initial dose of 175 mg every 4 weeks, with dose escalation to 400 mg IT every 2 weeks. Administration of IV HPβCD was associated with a substantial reduction in liver size, compared to expected volume for age and weight[56], and improvements in transaminase levels. See **Table 1.**  The patient experienced multiple episodes of status epilepticus requiring inpatient hospitalizations following IT HPβCD, although this type of event was also reported prior to the start of therapy. As the increased seizure activity was most pronounced immediately following the higher IT dose (400 mg) the dose was subsequently decreased to 300 mg, and indeed no subsequent seizure activity was seen at this stable lower dose. Her laboratory studies including complete blood counts, chemistry, lipid and coagulation panels remained stable. Her clinical severity scores improved and stabilized during the course of this intervention, with the exception of the seizure score due to difficulty in controlling. IV administration was transitioned to the home environment without drug related adverse events. The patient remained clinically stable on sequential therapy with some global improvements noted including the ability to sit unassisted, take sips of liquids, and tolerate up to 1 hour off the ventilator per day. Her liver size normalized for age. She experienced seven SAEs for status epilepticus and one SAE for a Port-a-Cath malfunction requiring device revision.

Patient **SEQ 9** is a Caucasian female who was diagnosed at 20 years of age following development of an ataxic gait in the context of neurocognitive decline. She had a long history of developmental delay and learning difficulties that, in retrospect, date back to age 5 years of age but became noticeably problematic in middle school. She attended school and was able to keep up with her peers and attain good grades until approximately age 15 years. For her last 4 years of school (high school) she required increasing assistance and then eventually special education assistance for all her classes. She was not able to pass a high school exit examination. She was never able to ride a bike and often assumed unusual body positions. She developed fine and gross motor control difficulties in her late teens with an ataxic gait and frequent falls, and this in combination with her unexplained cognitive decline, led to a neurological evaluation and eventually the diagnosis of *NPC1*. Diagnosis was confirmed with positive cultured skin fibroblast filipin staining and confirmed with genetic analysis. Imaging showed her liver to be mildly enlarged as well as the presence of right lower lobe lung opacities, consistent with prior aspiration. She presented with mild to moderate sensorineural hearing loss and hearing aids. She initiated IV HPβCD (Kleptose®) at age 24 years, at a dose of 2500 mg/kg weekly over 8 hours, and this has been transitioned to every 2 weeks at home. She did occasionally experience nausea or emesis following the IV administration and this was subsequently prevented with hydration and anti-emetics. After 4 weeks of infusions, IT HPβCD was started at a dose of 350 mg every 2 weeks. She experienced occasional nausea and emesis following the infusion, which was easily managed with fluids, rest and anti-emetics. She has received HPβCD for almost 2 years and her neurocognitive decline has stabilized with waxing and waning of memory, gait, and swallowing and more frequent falls. Her clinical severity score has been stable since initiation of IV and IT infusions. She has not experienced any serious adverse events of therapy. She had a lumbar port placed for ease of administration of CNS directed therapy.

***1.2 IV treatment alone with HPβCD***

Out of the 12 patients described herein, three individuals have received IV treatment only (IV1 with Kleptose® and IV2 and IV3 with Trappsol® Cyclo™).

Patient **IV1** is a Caucasion female diagnosed at 8 months of age by abnormal cultured skin fibroblast filipin staining, and later confirmed to be *NPC1* by molecular diagnostics. She had neonatal jaundice with abdominal distention and at age 2 months she was determined to have hepatosplenomegaly and ascites. Liver biopsy at 5 months demonstrated hepatic fibrosis. By age 3 years she had residual asymptomatic organomegaly. She walked at 18 months and had early global developmental delay, requiring speech therapy. She attended early grade school though her development regressed significantly following her first seizure at 8 years of age. Her seizures were difficult to control and in a short time span (6 months) she lost the ability to ambulate, speak or swallow. A gastrostomy tube was placed for feeding. She developed her first of many recurrent episodes of aspiration pneumonia at age 10 years, and neurogenic scoliosis and joint contractures developed soon thereafter. Due to recurrent aspiration the gastrostomy was revised to a gastrojejunostomy tube, and following a significant episode requiring intubation and ventilation, she required placement of a tracheostomy. At the time of initiation of IV HPβCD (Kleptose®) at age 18 years she had developed spastic quadriplegia, was ventilator dependent, non-ambulatory and non-verbal, enterally fed, and had a refractory seizure disorder. Her IV dose was initiated at 500 mg/kg weekly and escalated by 500 mg/kg monthly to 2000 mg/kg weekly. She has transitioned to home IV infusions due to lack of safety concerns. She has had multiple hospitalizations not attributable to medication which include: fevers, tracheitis, Port-a-Cath infection (*Pseudomonas putida*) eventually requiring removal, treatment with antibiotics, subsequent revision and re-infection, as well as worsening seizures, pyelonephritis, metabolic acidosis, proteinuria and hypertension of unknown etiology. Possible medication related adverse events include elevated transaminases and proteinuria, as well as the Port-a-Cath infections considered related to protocol device. She received IV treatment 17 months and her clinical course remained relatively stable (though severely affected) with respect to progression of NPC.

Patient **IV2** is a Brazilian male who was diagnosed with NPC at 15 years of age. Notable medical history includes hepatosplenomegaly, dystonia, and VSGP. Treatment with IV Trappsol® Cyclo™ started in April 2013, using an initial dose of ~1700 mg/kg every week. Safety data was collected for a period of 26 months of treatment with Trappsol® Cyclo™. The patient experienced two instances of rash which were effectively controlled by standard medical care. The additional medically concerning AEs that the patient experienced were consistent with the natural history of NPC. Limited information on the NCSS is available on this patient.

Patient **IV3** is a Brazilian female who was first diagnosed with NPC when she was 16 years old. The patient was diagnosed with VSGP followed by dysphagia and dysarthria. The patient started IV treatment with Trappsol® Cyclo™ using an initial dose of ~2600 mg/kg. Safety data has been reviewed for 32 months of IV treatment. Based upon the limited information available, it appears that the patient remained clinically stable during the course of treatment with Trappsol® Cyclo™. Initial treatment was associated with two adverse reactions, namely vomiting, tremor, chills, and fever, which were effectively controlled by standard medical care. No subsequent AEs were recorded for this patient and no clinical outcomes data were reported.
